# Supplementary material for: Theoretical Evaluation of Sulfur-Based Reactions as a Model for Biological Antioxidant Defense
Source: Int J Mol Sci. 2022 Nov 22;23(23):14515. doi: 10.3390/ijms232314515 (PMC9741100; doi:10.3390/ijms232314515)
Supplement: Supplementary file 1 [file ijms-23-14515-s001.zip › ijms-1997706-supplementary.pdf]

SUPPORTING INFORMATION

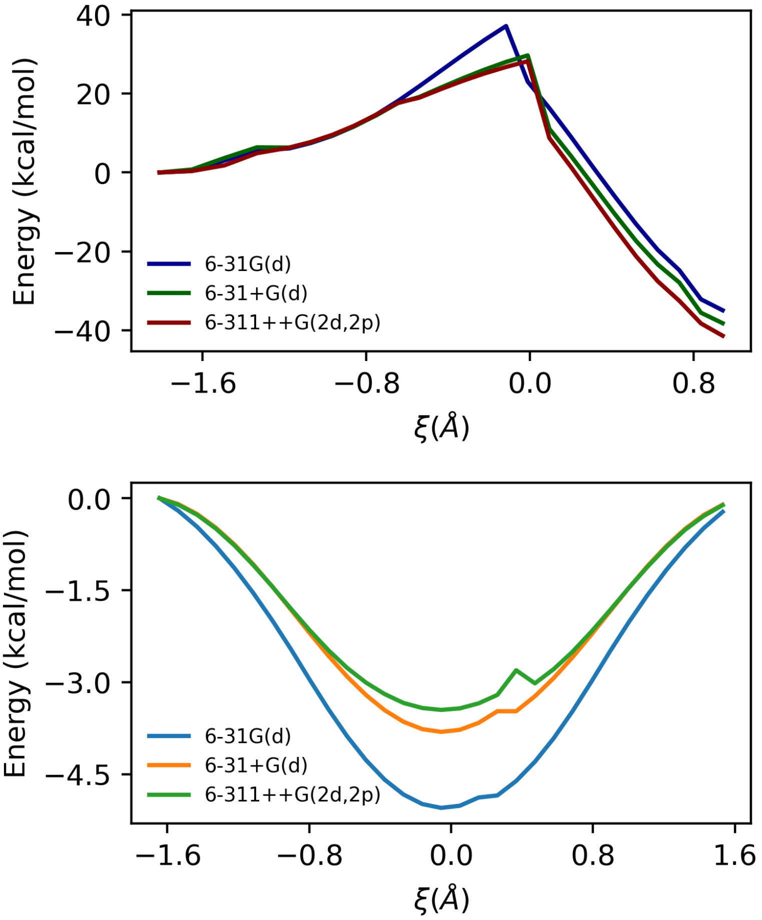

Figure S1: Upper panel: Relative energy associated to ethyl methyl sulfide oxidation by H_2O_2. Bottom panel: effect of the basis set with an increasing number of diffuse functions on the energy profile for the thiol/disulfide exchange reaction.

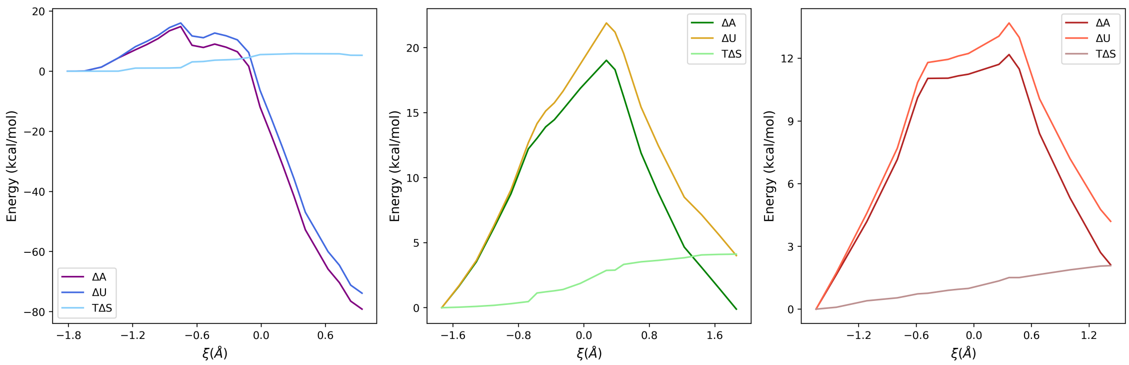

Figure S2: Internal energy, free energy and entropy (expressed as TΔS) variation along the reaction coordinate for Met oxidation in water (*left*), mercaptoethanol in water (*middle*) and butanethiol in DMF (*right*).

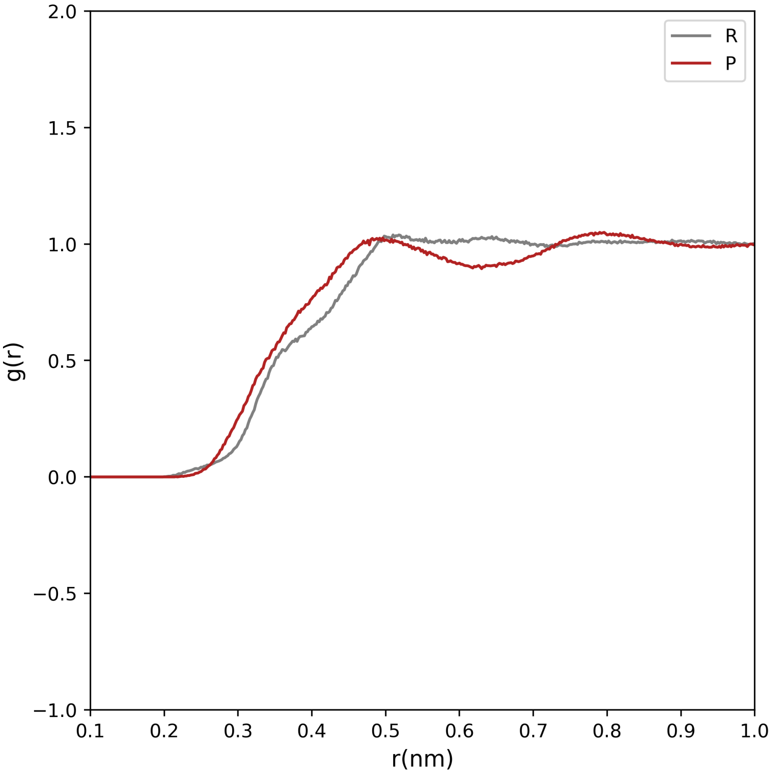

Figure S3: Sulfur-oxygen radial distribution function for Methionine (R) and Methionine sulfoxide (P) in water. The maximum of the function at ≈ 5.5 Å was chosen to select the water molecules belonging to the first solvation shell.

Table S1: ESP atomic charges (B3LYP/6-311++G(2d,2p)) of the three electronegative centers involved in the investigated reactions. Their structures and the corresponding MOs are reported in Figure 2. Partial atomic charges are reported in atomic units.

	Atom	ESP partial charges (a.u.)					
scan point		point 1	point 2	point3	point 4	point 5	point 6
Sulfide oxidation	S	-0.21	-0.30	-0.08	0.07	0.03	0.25
	O_P	-0.42	-0.25	-0.25	-0.30	-0.36	-0.40
	O_D	-0.38	-0.42	-0.57	-0.75	-0.65	-0.83
Disulfides exchange	S_{Nu}	-0.89	-0.78	-0.61	-0.56	-0.42	-0.28
	S_C	-0.03	0.06	0.08	0.08	0.06	0.02
	S_{LG}	-0.26	-0.43	-0.61	-0.67	-0.80	-0.87
		Reaction coordinate (Å)					
ξ_{SO}		-1.81	-1.07	0.54	0.01	0.09	0.9
ξ_{SS}		-1.64	-1.01	-0.16	0.26	0.68	1.43

In Table S1 with ξ_{SO} and ξ_{SS} we referred to the reaction coordinate for Met oxidation and thiol/disulfide exchange reaction, respectively.

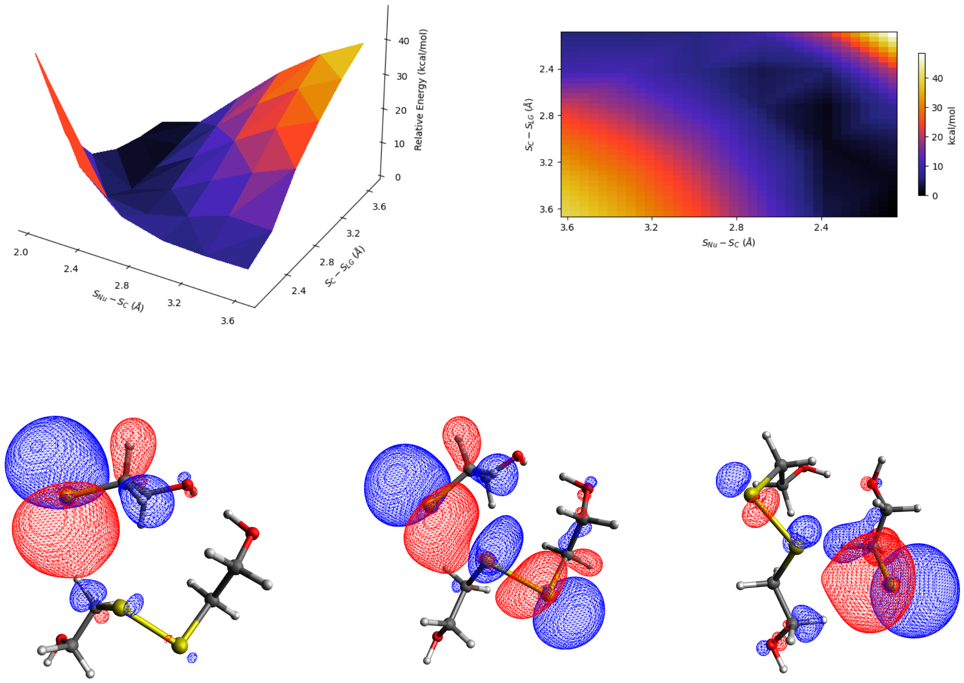

Figure S4: Upper panel: from left to right, the 3D and 2D PES scan for ME system (minimum at ξ = -0.573 $\mathring{A}$). Bottom panel: HOMO for reactants, TS and product state, calculated at the B3LYP/6-311++G(2d,2p) level of theory.

Table S2: ESP atomic charges (B3LYP/6-311++G(2d,2p)) of the three sulfur atoms for ME calculated in six points along the reaction coordinate.

	Atom	ESP partial charges (a.u.)					
scan point		point 1	point 2	point3	point 4	point 5	point 6
Disulfides exchange	S_{Nu}	-0.85	-0.80	-0.51	-0.60	-0.35	-0.25
(ME)	S_C	-0.16	-0.18	-0.25	-0.24	-0.19	-0.09
	S_{LG}	-0.26	-0.35	-0.65	-0.58	-0.80	-0.86
		Reaction coordinate (Å)					
ξ_{SS}		-1.74	-1.10	-0.36	-0.04	0.91	1.65
